# Replacement of the Double Meropenem Disc Test with a Lateral Flow Assay for the Detection of Carbapenemase-Producing Enterobacterales and *Pseudomonas aeruginosa* in Clinical Laboratory Practice

**DOI:** 10.3390/antibiotics12040771

**Published:** 2023-04-17

**Authors:** Areti Tychala, Georgios Meletis, Paraskevi Mantzana, Angeliki Kassomenaki, Charikleia Katsanou, Aikaterini Daviti, Lydia Kouroudi, Lemonia Skoura, Efthymia Protonotariou

**Affiliations:** Department of Microbiology, AHEPA University Hospital, School of Medicine, Aristotle University of Thessaloniki, S. Kiriakidi Str. 1, 54636 Thessaloniki, Greece; aretich@gmail.com (A.T.);

**Keywords:** *Klebsiella pneumoniae*, *Pseudomonas aeruginosa*, carbapenemases, NDM, KPC, IMP, VIM, OXA-48, LFA

## Abstract

The prompt detection of carbapenemases among Gram-negative bacteria isolated from patients’ clinical infection samples and surveillance cultures is important for the implementation of infection control measures. In this context, we evaluated the effectiveness of replacing phenotypic tests for the detection of carbapenemase producers with the immunochromatographic Carbapenem-Resistant K.N.I.V.O. Detection K-Set lateral flow assay (LFA). In total, 178 carbapenem-resistant Enterobacterales and 32 carbapenem-resistant *Pseudomonas aeruginosa* isolated in our hospital were tested with both our established phenotypic and molecular testing procedures and the LFA. The Kappa coefficient of agreement for Enterobacterales was 0.85 (*p* < 0.001) and 0.6 (*p* < 0.001) for *P. aeruginosa*. No major disagreements were observed and notably, in many cases, the LFA detected more carbapenemases than the double meropenem disc test, especially regarding OXA-48 in Enterobacterales and VIM in *P. aeruginosa*. Overall, the Carbapenem-Resistant K.N.I.V.O. Detection K-Set was very effective and at least equivalent to the standard procedures used in our lab. However, it was much faster as it provided results in 15 min compared to a minimum of 18–24 h for the phenotypic tests.

## 1. Introduction

β-lactam antibiotics are widely used in medical practice because of their effectiveness and their limited adverse effects [1]. They share a common β-lactam ring and act by binding to and inactivating the penicillin-binding proteins (PBPs), thus inhibiting bacterial cell wall formation. This category includes penicillins, cephalosporins, monobactams, and carbapenems, which are the most effective among the β-lactams and are less susceptible to the mechanisms of acquired bacterial resistance [2]. Therefore, the emergence and spread of carbapenem resistance is considered of major importance for public health [3]. Even though carbapenem resistance may be multi-factorial [4], the major resistance determinant against carbapenems among Gram-negative nosocomial pathogens is the production of enzymes able to hydrolyze these agents together with other β-lactams. These enzymes are commonly encoded by genes that are harbored in mobile genetic elements capable of horizontal gene transfer and consequent rapid dissemination [5].

Generally, all enzymes with the potential to hydrolyze at least some β-lactam antibiotics are called β-lactamases [6]. β-lactamases are categorized into four distinct molecular classes according to the Ambler classification [7]; class A, C, and D enzymes have serine in their active center, whereas class B have zinc in their active center and are, therefore, called metallo-beta-lactamases (MBLs). Enzymes of all classes that are able to hydrolyze carbapenems together with other β-lactam antibiotics are called carbapenemases [8,9]. Among class A enzymes, *Klebsiella pneumoniae* carbapenemases (KPCs) [10,11] are the most clinically important and have spread worldwide. Similarly, among class D representatives, oxacillinase-48 (OXA-48) and OXA-48-like enzymes have a wide global distribution [12]. Class B [13] includes, among other carbapenemases, imipenemases (IMPs) [14], Verona integron-encoded metallo-β-lactamases (VIMs) [15], and New Delhi metallo-β-lactamases (NDMs) [16]. Class C enzymes are not considered carbapenemases; some of them, however, may present a low potential of carbapenem hydrolysis and their overproduction may contribute to carbapenem resistance combined with diminished outer membrane permeability and/or efflux pump over-expression [17]. Overall, the most effective carbapenemases, in terms of carbapenem hydrolysis and geographical spread, are KPC, VIM, IMP, NDM, and OXA-48 types [18].

KPCs present with some specific characteristics. They inactivate all β-lactam antibiotics, are only partially inhibited by older β-lactamase inhibitors, such as clavulanic acid, tazobactam, and boronic acid, and are commonly inhibited by novel inhibitors, such as avibactam, relebactam, and vaborbactam. In phenotypic tests they are inhibited by phenylboronic acid but not ethylene diamine tetraacetic acid (EDTA). MBLs, on the other hand, can hydrolyze all β-lactams except aztreonam and cefiderocol, whereas they are not inhibited by the aforementioned β-lactamase inhibitors or by phenylboronic acid. Since they bear zinc in their active center, their in vitro inhibition is achieved by metal chelators, such as EDTA. Among them, NDM-type enzymes present unique features. They hydrolyze aztreonam and they commonly present negative modified Hodge test results [19].

The rapid and accurate identification of carbapenemase production among Gram-negative isolates recovered by patient infections or surveillance cultures is important for both therapeutic and infection control purposes. Several culture and non-culture based diagnostic tests have been developed. Non-culture methods include the molecular detection of carbapenemase-related genes, the biochemical detection of carbapenem hydrolysis, and antibody-based methods such as lateral flow immunoassays (LFA) for carbapenemase identification. Molecular assays such as multiple polymerase chain reaction (PCR) and loop-mediated isothermal amplification (LAMP) can rapidly detect and discriminate between different types of carbapenemase genes. Nevertheless, such methods require special equipment and are expensive. On the contrary, a lateral flow immunoassay (LFA) is a simple, rapid, and relatively low-cost method that could be used instead of the above expensive examples. Recently, several lateral flow assays have been introduced worldwide for the rapid and easy detection of multiple carbapenemases, substantially reducing the turnaround time compared to conventional culture-based methods [20].

Therefore, we evaluated the effectiveness of replacing phenotypic assays for the detection of carbapenemase producers with the new Carbapenem-Resistant K.N.I.V.O. Detection K-Set multiplex lateral flow assay developed by Goldstream, Beijing Gold Mountain river Tech Development Co., Ltd. (Beijing, China). For this purpose, we tested carbapenem-resistant Enterobacterales and *Pseudomonas aeruginosa* isolated in our hospital with both our established phenotypic and molecular testing procedures and the LFA.

## 2. Results

**Figure 1:**
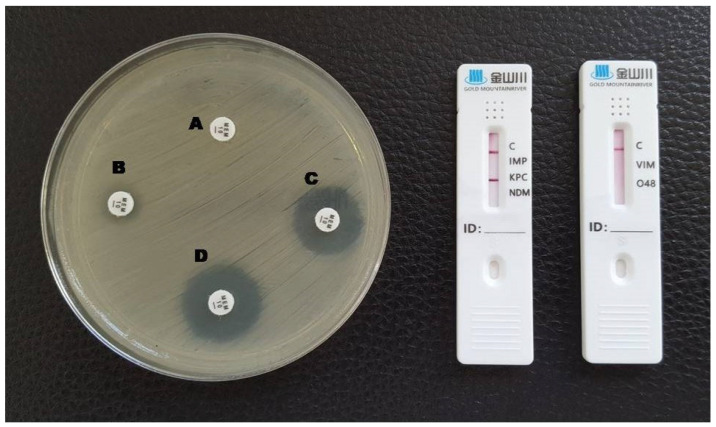
Phenotypic and lateral flow assay results for a KPC-producing *K. pneumoniae*. A: meropenem disc without inhibitors; B: meropenem + EDTA; C: meropenem + phenylboronic acid; D: meropenem + EDTA + phenylboronic acid. A red line in the KPC test area is visible in the LFA.

**Figure 2:**
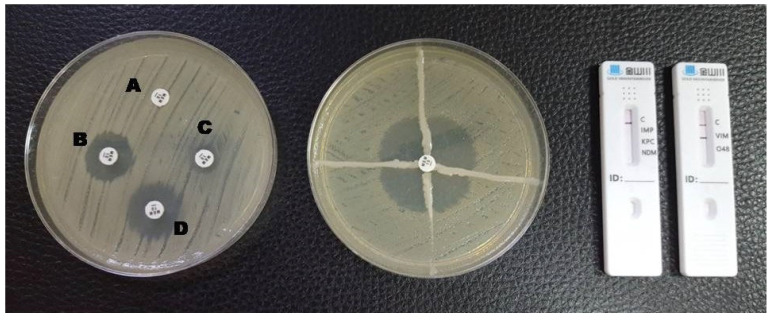
Phenotypic and lateral flow assay results for a VIM-producing *K. pneumoniae*. A: meropenem disc without inhibitors; B: meropenem + EDTA; C: meropenem + phenylboronic acid; D: meropenem + EDTA + phenylboronic acid. The Hodge test was positive and a red line in the VIM test area is visible in the LFA.

**Figure 3:**
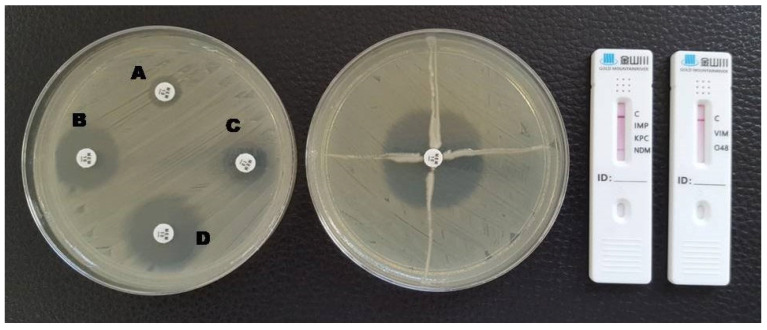
Phenotypic and lateral flow assay results for an NDM-producing *K. pneumoniae*. A: meropenem disc without inhibitors; B: meropenem + EDTA; C: meropenem + phenylboronic acid; D: meropenem + EDTA + phenylboronic acid. The Hodge test was negative and a red line in the NDM test area is visible in the LFA.

**Figure 4:**
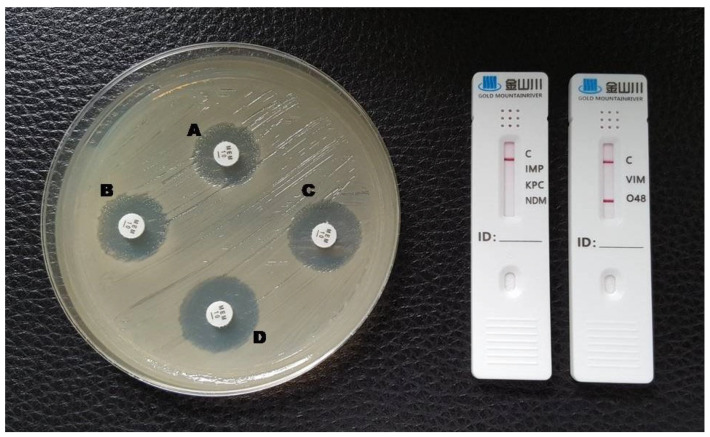
Phenotypic and lateral flow assay results for an OXA-48-producing *K. pneumoniae*. A: meropenem disc without inhibitors; B: meropenem + EDTA; C: meropenem + phenylboronic acid; D: meropenem + EDTA + phenylboronic acid. The test could not be interpreted by the double meropenem disc test (indeterminate) and a red line in the O48 test area is visible in the LFA.

In total, 210 single patient isolates were tested using both the double meropenem disc test (DMDT) and the K.N.I.V.O. Detection K-Set LFA. Among the studied isolates, 178 were Enterobacterales (155 *K. pneumoniae*, 10 *Proteus mirabilis,* 9 *Providencia stuartii*, 2 *Escherichia coli*, 1 *Klebsiella oxytoca,* and 1 *Enterobacter cloacae* complex) and 32 were *Pseudomonas aeruginosa*. The Enterobacterales were isolated from blood cultures (n = 67), urine samples (n = 40), bronchoalveolar secretions (n = 30), wound samples (n = 6), central venous catheters (n = 9), pleural fluid (n = 1), pus samples (n = 1), and rectal swabs (n = 24) taken for surveillance purposes at patient admission or during hospitalization. *P. aeruginosa* isolates were recovered from 18 blood cultures (n = 18), 5 urine samples (n = 5), bronchoalveolar secretions (n = 3), soft tissue infections (n = 1), 2 central venous catheters (n = 2), pleural fluid (n = 1), pus samples (n = 1), and rectal swabs (n = 1). KPC, OXA-48, and MBL including VIM and NDM were detected among the Enterobacterales as shown in Supplementary Table S1 and Figure 1, Figure 2, Figure 3 and Figure 4. The Kappa coefficient of agreement between the two methods was 0.85 (*p* < 0.001).

**Figure 5:**
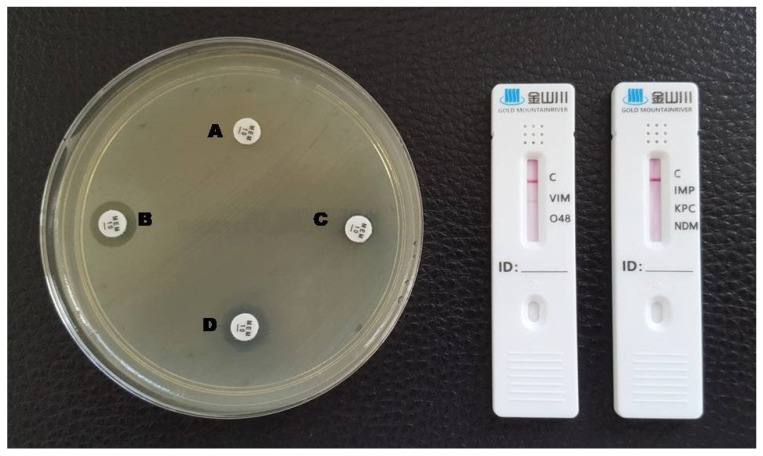
Phenotypic and lateral flow assay results for a VIM-producing *P. aeruginosa*. A: meropenem disc without inhibitors; B: meropenem + EDTA; C: meropenem + phenylboronic acid; D: meropenem + EDTA + phenylboronic acid. A red line in the VIM test area is visible in the LFA.

*P. aeruginosa* isolates were found to produce only the VIM carbapenemase (Supplementary Table S2 and Figure 5). The Kappa coefficient of agreement between the two methods for *P. aeruginosa* was 0.6 (*p* < 0.001).

## 3. Discussion

In recent years, rapid diagnostic testing using LFAs for the detection of enzymes associated with antimicrobial resistance have been widely developed as they demonstrate comparable performance with gold standard PCR-based methods and can be easily applied in clinical microbiology laboratories without requiring specialized personnel and excessive cost [21,22,23,24,25].

In our study, the performance of K.N.V.I.O. for Enterobacterales was very good since the Kappa coefficient for Enterobacterales showed a strong agreement between the two methods. In fact, the level of agreement could have been even better; however, the presence of OXA-48 carbapenemases that were detected by the LFA and not detected by the DMDT biased the Kappa coefficient result. Indeed, the presence of OXA-48 cannot be detected by the DMDT; thus, the respective result is commonly indeterminate and cannot be interpreted. Of interest, there were no major disagreements between the two methods (for KPC instead of MBL) that could imply failures in the performance of the LFA. Moreover, in many cases, the LFA detected more carbapenemases than the DMDT could have done.

The Kappa coefficient for *P. aeruginosa* showed moderate agreement and this is exclusively because of the limitations of the DMDT to accurately detect carbapenemases in this species. Carbapenem resistance in *P. aeruginosa* might be more influenced than in other clinically important species by additional mechanisms such as efflux pumps over-expression or porin loss [26]. This may explain the inability of the DMDT to detect the presence of carbapenemases, because the interpretation of the test is based on comparing the inhibition halo of meropenem with and without the presence of specific inhibitors, especially if the carbapenemase is expressed at low levels. On the other hand, LFAs do not present the same limitation, and this can explain the detection of the VIM carbapenemase in isolates where the DMDT was not able to detect them.

Overall, in our study the performance of the LFA was satisfactory and improved the turnaround times of our laboratory. First, the use of the LFA allowed for the immediate distinction between VIM and NDM metallo-β-lactamases without the need to perform the Hodge test and PCR. Specifically, the use of the Hodge test is often problematic because it frequently produces an uncertain interpretation and can be biased by the presence of additional carbapenemases. Second, the LFA, in some cases, detected more carbapenemases than the DMDT in Enterobacterales, including OXA-48. Specifically, the presence of OXA-48 in combination with other carbapenemases may often bias the interpretation of the DMDT. Third, the presence of OXA-48 cannot be detected by the DMDT even when no other carbapenemases are present and the use of the LFA rules out this limitation.

In practical terms, the superiority of the LFA is overwhelming. It is much easier to perform than the DMDT and gives results in 15 min for the five major carbapenemases. Of note, the DMDT provides results after 18–24 h of incubation.

Josa et al. showed that LFAs outweigh phenotypic boronic acid and EDTA synergy tests in carbapenemase detection and differentiation for Enterobacterales and *P. aeruginosa*. That is expected since the synergy tests can only detect MBLs, with no discrimination among them, and KPCs, but not OXA-48-like carbapenemases or any combination of them. Additionally, they may present false-positive results as EDTA may affect membrane permeability and boronic acid may increase the zone of inhibition of meropenem > 5 mm in case of AmpC hyper-production [27]. Sadek et al. found the K.N.I.V.O. Detection K-Set to have excellent performance, with 96.8% sensitivity and 100% specificity against a collection of 252 well-characterized Gram-negative strains. In this study, it succeeded in identifying KPC, NDM, IMP, VIM, and OXA-48-like carbapenemases with the exception of IMP-2, -8, -13, -19, which are mostly reported in Asian countries and only sporadically in Europe [28].

Carbapenem resistance is considered a public health issue of utmost importance as it is implicated in prolonged hospital stay and increased morbidity and mortality in hospitalized patients. The changing epidemiology and wide spread of different carbapenemases impedes their detection through culture-based methods, which are considered inadequate nowadays. Precision medicine practices, through the accurate identification of carbapenemases, are essential in order to promptly and efficiently apply infection control measures and therapeutics.

Carbapenemase-producing Gram-negative bacilli may occur by selective suppression on patient’s flora, patient-to-patient transmission, or both. This, alongside plasmid transmission between species, makes them rapidly disseminate in the hospital environment and persist there as they are difficult to treat and eradicate. Considering the divergence of carbapenemases, speed and accuracy in their tracking is crucial in order to rapidly identify potential outbreaks, apply infection control measures to withhold their spread, and avoid unnecessary isolation.

Furthermore, in the past few years, new inhibitor/b-lactam combinations have been developed and are included in the current guidelines for the treatment of MDR Gram-negative bacteria [29], whereas their activity is dependent on their mechanisms of resistance. Therefore, knowing the exact type of the carbapenemase present is a prerequisite for the proper use of these molecules. For instance, ceftazidime–avibactam is active against extended spectrum β-lactamase producers, non-MBL carbapenem-resistant *P. aeruginosa*, KPC-producing carbapenem-resistant Enterobacterales, and OXA-48-producing carbapenem-resistant Enterobacterales, but not against MBL-producing Enterobacterales and MBL-producing *P. aeruginosa*. Furthermore, infections by carbapenem-resistant Gram-negative bacteria are associated with high mortality rates and thus the prompt administration of the proper antimicrobial regiment is crucial [30].

In conclusion, the characterization of specific carbapenemases is crucial and, therefore, the double synergy test, which is widely used in Greece, does not seem appropriate in an endemic and highly diverse environment such as ours. Thus, we also suggest that the local epidemiology should be taken into account when selecting the most suitable test for carbapenemase detection.

Knowledge of the local epidemiology plays an important role in the practical implementation of tests. For example, apart from carbapenemase-production, *P. aeruginosa* is known to show resistance to carbapenems due to porin defects and this can influence the interpretation of phenotypic tests. In our area, however, which is endemic for VIM-producing *P. aeruginosa*, carbapenem resistance in this species is common due to carbapenemases; thus, carbapenem-resistant isolates resulting from porin defects alone are rare.

Even though we included only single patient isolates, no sequencing-based or other molecular epidemiology methods were employed to better characterize the molecular epidemiology of the isolates, since this was beyond the scope of this work. This is a limitation of our work because the possible presence of a multi-drug-resistant clone in our collection (especially regarding VIM-producing *P. aeruginosa*) cannot be definitely excluded. Another limitation is that we only employed a molecular “gold-standard” method for the isolates that presented discrepancies between the results of the phenotypic methods and the LFA. Indeed, in some Enterobacterales in our collection (4/14) that were tested with the molecular technique, the Antimicrobial Resistance Direct Flow Chip detected the presence of *bla_NDM_*, whereas the respective LFA result was negative for the NDM carbapenemase, indicating that the LFA lacks sensitivity as compared with the molecular gold standard. Our results are indicative of the performance of a single LFA kit and should not be generalized for all other available kits. Therefore, we would like to recommend the evaluation of LFAs for carbapenemase detection before their implementation in clinical practice.

## 4. Materials and Methods

### 4.1. Study Design

A total of 210 carbapenem-resistant Gram-negative bacteria (Enterobacterales and *P. aeruginosa*) were tested in parallel using the double meropenem disc-test [31] and the Carbapenem-Resistant K.N.I.V.O. Detection K-Set (Goldstream, Beijing Gold Mountain river Tech Development Co., Ltd., Beijing, China) immunochromatographic lateral flow assay. The isolates were recovered from patients hospitalized in AHEPA University Hospital between September 2021 and September 2022. Bacterial identification and antimicrobial susceptibility testing were performed using the automated system VITEK2 (bioMérieux, Marcy l’Etoile, France). Susceptibility testing results were interpreted according to the EUCAST breakpoints v 12.0 (2022). All isolates were tested phenotypically for the detection of MBL, KPC, or both carbapenemase categories using the meropenem disc test. Based on our hospital’s epidemiology, MBL-positive *K. pneumoniae* were considered as probable VIM or NDM producers [32]. Therefore, MBL-positive *K. pneumoniae* isolates were further tested with the modified Hodge test [33]. In case of a negative Hodge test result, the isolate was considered as a probable NDM producer and was further tested using PCR for the detection of the *bla*_NDM_ gene. In cases of DMDT double positivity (positive for both MBL and KPC), the Hodge test was not performed for distinguishing VIM and NDM because it would be influenced by the presence of KPC. According to the local epidemiology, MBL-positive *P. mirabilis* and *P. aeruginosa* were deemed to be probable VIM producers [34,35] and were not tested further using the Hodge test or PCR. The discrepancies between the phenotypic tests and the LFA were resolved using the Antimicrobial Resistance Direct Flow Chip (AMR): a microarray-based molecular diagnostic assay (Master Diagnóstica Granada, Spain). This molecular assay is able to detect KPC, NDM, IMP, various OXA-type carbapenemases, GES, GIM, NMC/IMI, SME, SIM, CMY, DHA, CTX, SHV, and SIM β-lactamases.

### 4.2. Double Meropenem Disc Test

The double meropenem disc test is a disc test where four meropenem discs are used with and without carbapenemase inhibitors (EDTA and phenylboronic acid) [31]. First, a 0.5 McFarland bacterial suspension was prepared and bacteria were inoculated onto a Mueller Hinton agar plate. The meropenem discs were placed on the surface of the agar preferably in a cross-like formation. The first disc was left without inhibitors. In total, 10 μL of EDTA 0.1 M were added on the second and 20 μL (20 g/L) of phenylboronic acid were added on the third disc. On the fourth disc, both inhibitors were applied. The evaluation of the results was performed after 18–24 h of incubation as follows: No inhibition zone around the first disc or an inhibition zone with a diameter of <22 mm for Enterobacterales or <14 mm for *P. aeruginosa* is indicative of carbapenem resistance. An inhibition zone around the second and the fourth disc with a diameter ≥ 5 mm that of the first disc indicates MBL production. An inhibition zone around the third and the fourth disc with a diameter ≥ 5 mm that of the first disc indicates KPC production. An inhibition halo around the second and third disc with a diameter ≥ 5 mm that of the first disc and an even larger halo around the fourth disc indicates simultaneous MBL and KPC production.

In our area, where many carbapenemase-encoding genes are endemic and carbapenem resistance is common due to the presence of carbapenemases, we usually consider a test negative when no carbapenemase is present. In such cases, the inhibition zone around the discs is larger than the breakpoint used and no further testing is needed. In the present work, the term “indeterminate” was used for tests where, despite the presence of carbapenem resistance, no information about the type of carbapenemase(s) present could be obtained. This commonly happens in the presence of OXA-type carbapenemases and further testing is needed.

Of note, EDTA can have intrinsic activity against bacteria by disrupting their cell wall and this can sometimes result in inhibition diameters larger than 5 mm for the EDTA-containing discs. To detect this, a negative control can be used by placing a blank disc with EDTA only. In this work, blank discs were not applied because the test has been used for many years in our lab and we have noticed that this property of EDTA does not influence the interpretation of results among our isolates that may carry specific carbapenemase-encoding genes, especially when these are not OXA-type carbapenemase determinants.

### 4.3. Modified Hodge Test

The modified Hodge test [36] was performed by inoculating the study isolate together with a carbapenem-susceptible indicator strain (*E. coli* ATCC 25922). Briefly, a 0.5 McFarland suspension of the indicator strain was inoculated onto a Mueller Hinton agar plate with a sterile cotton swab. Thereafter, a carbapenem disc was placed at the center of the plate. In total, 3–5 colonies of the test isolate were streaked from the center to the periphery of the plate. After incubation for 18–24 h, the presence of a distorted inhibition zone due to growth of the indicator strain toward the carbapenem disc because of carbapenemase production of the study isolate was interpreted as a positive result.

### 4.4. Detection of bla_NDM_ by PCR

The *bla*_NDM_ gene was detected by PCR as previously reported [37]. This method was applied for MBL-positive and Hodge-test-negative *K. pneumoniae* isolates, as identified via phenotypic methods. The primers used were NDM-Fm (5′-GGTTTGGCGATCTGGTTTTC-3′) and NDM-Rm (5′-CGGAATGGCTCATCACGATC-3′). The thermal cycling conditions used were 10 min at 94 °C; 36 cycles for 30 s at 94 °C, 40 s at 52 °C, and 50 s at 72 °C; concluding with 5 min at 72 °C for the final extension.

### 4.5. Carbapenem-Resistant K.N.I.V.O. Detection K-Set

The Carbapenem-Resistant K.N.I.V.O. Detection K-Set (Goldstream, Beijing Gold Mountain river Tech Development Co., Ltd.) is a multiplex lateral immunochromatographic flow assay that directly identifies carbapenemases from a bacterial colony. The assay consists of two cassettes: A and B. Cassette A includes specific areas for the detection of VIM and OXA-48, whereas cassette B includes areas for IMP, KPC, and NDM detection. Both cassettes include a specific control (C) area. In case the control line does not appear, the test result should be considered invalid. If only one line appears in the control region, the sample is considered negative since it does not contain any carbapenemase or may contain carbapenemase(s) at a non-detectable level. The result is interpreted as positive if the red line appears in the control region and one or several lines appear in the VIM, O48 (OXA-48), IMP, KPC, and NDM test regions. The intensity of the red test lines may vary; thus, a weak line still indicates a positive result since it may represent cases of lower enzyme expression. The assay was performed according to the manufacturer’s instructions. In total, 2–3 single isolated colonies of the isolate to be tested were collected from the plate with an inoculation loop and were resuspended in an Eppendorf tube containing the five drops of sample treatment solution included in the kit. Subsequently, 50 μL of the mixture were added horizontally into the sample well of each cassette. The results were interpreted visually after 10–15 min of incubation at room temperature.

### 4.6. Statistical Analysis

The Kappa coefficient of agreement was applied to assess the level of agreement between the DMDT and the LFA results in Enterobacterales and in *P. aeruginosa* isolates using SPSS 28.0.

## 5. Conclusions

The Carbapenem-Resistant K.N.I.V.O. Detection K-Set was equivalent to the standard procedures used in our lab for the detection of carbapenamases; however, it was much faster since it provided results in 15 min compared to a minimum of 24 h the aforementioned methods. Therefore, it is a valuable tool in the early implementation of appropriate antimicrobial therapy and infection control measures.

## Data Availability

The data presented in this study are available in the article and the supplementary material.

## Supplementary Materials

The following supporting information can be downloaded at: https://www.mdpi.com/article/10.3390/antibiotics12040771/s1, Table S1: Carbapenem-resistant Enterobacterales tested for carbapenemase production using the double meropenem disc test (DMDT), the Hodge test, the PCR for NDM, and the lateral flow assay (LFA); Table S2: Carbapenem-resistant *P. aeruginosa* isolates tested for carbapenemase production using the double meropenem disc test (DMDT) and the lateral flow assay (LFA).
