# Supplementary material for: Replacement of the Double Meropenem Disc Test with a Lateral Flow Assay for the Detection of Carbapenemase-Producing Enterobacterales and Pseudomonas aeruginosa in Clinical Laboratory Practice
Source: Antibiotics (Basel). 2023 Apr 17;12(4):771. doi: 10.3390/antibiotics12040771 (PMC10135102; doi:10.3390/antibiotics12040771)
Supplement: Supplementary file 1 [file antibiotics-12-00771-s001.zip › antibiotics-2305705-supplementary.docx]

**Table S1.** Carbapenem-resistant Enterobacterales tested for carbapenemase production using the double meropenem disc test (DMDT), the Hodge test, the PCR for NDM, and the lateral flow assay (LFA). The Antimicrobial Resistance Direct Flow Chip (AMR) was used in cases of disagreement. Br sec: bronchoalveolar secretions; CVC: central venous catheter; N/A: not applicable; NEG: negative; POS: positive; IN: indeterminate; MBL: metallo-β-lactamase; VIM: Verona integron-encoded metallo-β-lactamase; NDM: New Delhi metallo-β-lactamase; KPC: *Klebsiella pneumoniae* carbapenemase; OXA-48: oxacillinase-48.

| **Isolate ID** | **Species** | **Source** | **DMDT** | **HODGE** | **PCR for NDM** | **LFA** | **disagreement** | **AMR** |
| --- | --- | --- | --- | --- | --- | --- | --- | --- |
| D1248 | *K. oxytoca* | Blood | MBL | POS | N/A | VIM | NO | N/A |
| D1474 | *K. pneumoniae* | Blood | MBL | NEG | POS | NDM | NO | N/A |
| D1592 | *K. pneumoniae* | Blood | KPC | N/A | N/A | KPC | NO | N/A |
| D1728 | *K. pneumoniae* | Blood | MBL | NEG | POS | NDM | NO | N/A |
| D1778 | *K. pneumoniae* | Blood | KPC | N/A | N/A | KPC | NO | N/A |
| D1816 | *K. pneumoniae* | Blood | MBL | NEG | POS | NDM | NO | N/A |
| D1817 | *K. pneumoniae* | Blood | KPC | N/A | N/A | KPC | NO | N/A |
| D1843 | *K. pneumoniae* | Blood | KPC | N/A | N/A | KPC | NO | N/A |
| D1849 | *K. pneumoniae* | Blood | MBL | POS | N/A | NDM | NO | N/A |
| D1859 | *K. pneumoniae* | Blood | MBL | NEG | POS | NDM | NO | N/A |
| D1550 | *K. pneumoniae* | Blood | KPC | N/A | N/A | KPC | NO | N/A |
| D1186 | *K. pneumoniae* | Blood | KPC | N/A | N/A | KPC | NO | N/A |
| D1088 | *K. pneumoniae* | Blood | KPC | N/A | N/A | KPC | NO | N/A |
| D1163 | *K. pneumoniae* | Blood | MBL | NEG | POS | NDM | NO | N/A |
| D1404 | *K. pneumoniae* | Blood | MBL | NEG | POS | NDM | NO | N/A |
| D1436 | *K. pneumoniae* | Blood | MBL | NEG | POS | NDM | NO | N/A |
| D1622 | *K. pneumoniae* | Blood | KPC | N/A | N/A | KPC | NO | N/A |
| D2063 | *K. pneumoniae* | Blood | MBL | NEG | POS | NDM | NO | N/A |
| D2147 | *K. pneumoniae* | Blood | MBL | NEG | POS | NDM | NO | N/A |
| D2172 | *K. pneumoniae* | Blood | MBL | NEG | POS | NDM | NO | N/A |
| D2167 | *K. pneumoniae* | Blood | MBL | NEG | POS | NDM | NO | N/A |
| D135 | *K. pneumoniae* | Blood | MBL | NEG | POS | NDM | NO | N/A |
| D150 | *K. pneumoniae* | Blood | KPC | N/A | N/A | KPC | NO | N/A |
| D338 | *K. pneumoniae* | Blood | KPC | N/A | N/A | KPC | NO | N/A |
| D348 | *K. pneumoniae* | Blood | KPC | N/A | N/A | KPC | NO | N/A |
| D459 | *K. pneumoniae* | Blood | KPC | N/A | N/A | KPC | NO | N/A |
| D478 | *K. pneumoniae* | Blood | IN | N/A | N/A | OXA-48 | YES | OXA-48 |
| D594 | *K. pneumoniae* | Blood | KPC | N/A | N/A | KPC/NDM | NO | N/A |
| D700 | *K. pneumoniae* | Blood | KPC | N/A | N/A | KPC | NO | N/A |
| D720 | *K. pneumoniae* | Blood | KPC | N/A | N/A | KPC | NO | N/A |
| D759 | *K. pneumoniae* | Blood | KPC | N/A | N/A | KPC | NO | N/A |
| D770 | *K. pneumoniae* | Blood | KPC | N/A | N/A | KPC | NO | N/A |
| D785 | *K. pneumoniae* | Blood | KPC | N/A | N/A | KPC | NO | N/A |
| **Isolate ID** | **Species** | **Source** | **DMDT** | **HODGE** | **PCR for NDM** | **LFA** | **disagreement** | **AMR** |
| D811 | *K. pneumoniae* | Blood | KPC | N/A | N/A | KPC | NO | N/A |
| D843 | *K. pneumoniae* | Blood | MBL | NEG | POS | NDM | NO | N/A |
| D846 | *K. pneumoniae* | Blood | MBL | NEG | POS | NDM | NO | N/A |
| D861 | *K. pneumoniae* | Blood | MBL | NEG | POS | NDM | NO | N/A |
| D873 | *K. pneumoniae* | Blood | KPC | N/A | N/A | KPC | NO | N/A |
| D891 | *K. pneumoniae* | Blood | MBL | NEG | POS | NDM | NO | N/A |
| D935 | *K. pneumoniae* | Blood | KPC | N/A | N/A | KPC | NO | N/A |
| D933 | *K. pneumoniae* | Blood | KPC | N/A | N/A | KPC | NO | N/A |
| D1082 | *K. pneumoniae* | Blood | KPC | N/A | N/A | KPC | NO | N/A |
| D1137 | *K. pneumoniae* | Blood | KPC | N/A | N/A | KPC | NO | N/A |
| D1180 | *K. pneumoniae* | Blood | MBL | NEG | POS | NDM | NO | N/A |
| D1186 | *K. pneumoniae* | Blood | KPC/MBL | N/A | N/A | KPC/NDM | NO | N/A |
| D1213 | *K. pneumoniae* | Blood | MBL | NEG | POS | NDM | NO | N/A |
| D1195 | *K. pneumoniae* | Blood | KPC | N/A | N/A | KPC | NO | N/A |
| D1210 | *K. pneumoniae* | Blood | KPC | N/A | N/A | KPC | NO | N/A |
| B2094 | *K. pneumoniae* | Blood | KPC | N/A | N/A | KPC | NO | N/A |
| A4133 | *K. pneumoniae* | Blood | MBL | NEG | POS | NDM | NO | N/A |
| D1268 | *K. pneumoniae* | Blood | MBL | NEG | POS | NDM | NO | N/A |
| D1324 | *K. pneumoniae* | Blood | KPC | N/A | N/A | KPC | NO | N/A |
| D1342 | *K. pneumoniae* | Blood | MBL | NEG | POS | NDM | NO | N/A |
| D1391 | *K. pneumoniae* | Blood | KPC | N/A | N/A | KPC | NO | N/A |
| D1404 | *K. pneumoniae* | Blood | MBL | NEG | POS | NDM | NO | N/A |
| D1397 | *K. pneumoniae* | Blood | KPC/MBL | N/A | N/A | KPC/VIM | NO | N/A |
| D1398 | *K. pneumoniae* | Blood | KPC/MBL | N/A | N/A | KPC/VIM | NO | N/A |
| D1403 | *K. pneumoniae* | Blood | KPC | N/A | N/A | KPC | NO | N/A |
| D1955 | *K. pneumoniae* | Blood | KPC | N/A | N/A | KPC | NO | N/A |
| D1633 | *P. mirabilis* | Blood | MBL | NEG | POS | NDM | NO | N/A |
| D25 | *P. mirabilis* | Blood | MBL | POS | N/A | VIM | NO | N/A |
| D1180 | *P. mirabilis* | Blood | MBL | NEG | NEG | VIM | NO | N/A |
| D2049 | *P. stuartii* | Blood | IN | NEG | N/A | NEG | NO | N/A |
| D433 | *P. stuartii* | Blood | MBL | NEG | POS | NDM/VIM | NO | N/A |
| D480 | *P. stuartii* | Blood | MBL | NEG | NEG | VIM | NO | N/A |
| D1251 | *P. stuartii* | Blood | MBL | POS | N/A | NDM/VIM | NO | N/A |
| D1270 | *P. stuartii* | Blood | MBL | POS | N/A | VIM | NO | N/A |
| Β3149 | *K. pneumoniae* | Br sec | KPC | N/A | N/A | KPC | NO | N/A |
| Β3153 | *K. pneumoniae* | Br sec | MBL | NEG | POS | NDM | NO | N/A |
| Β3027 | *K. pneumoniae* | Br sec | KPC | N/A | N/A | KPC | NO | N/A |
| Β3418 | *K. pneumoniae* | Br sec | MBL | NEG | POS | NDM | NO | N/A |
| B3439 | *K. pneumoniae* | Br sec | KPC | N/A | N/A | KPC | NO | N/A |
| Β3447 | *K. pneumoniae* | Br sec | KPC | N/A | N/A | KPC | NO | N/A |
| B2702 | *K. pneumoniae* | Br sec | MBL | NEG | POS | NDM | NO | N/A |
| **Isolate ID** | **Species** | **Source** | **DMDT** | **HODGE** | **PCR for NDM** | **LFA** | **disagreement** | **AMR** |
| Β3929 | *K. pneumoniae* | Br sec | KPC | N/A | N/A | KPC | NO | N/A |
| Β3923 | *K. pneumoniae* | Br sec | KPC | N/A | N/A | KPC | NO | N/A |
| Β3970 | *K. pneumoniae* | Br sec | KPC | N/A | N/A | KPC | NO | N/A |
| Β4013 | *K. pneumoniae* | Br sec | KPC | N/A | N/A | KPC | NO | N/A |
| Β4011 | *K. pneumoniae* | Br sec | KPC | N/A | N/A | KPC | NO | N/A |
| Β4015 | *K. pneumoniae* | Br sec | KPC | N/A | N/A | KPC | NO | N/A |
| Β4046 | *K. pneumoniae* | Br sec | KPC | N/A | N/A | KPC | NO | N/A |
| Β203 | *K. pneumoniae* | Br sec | KPC | N/A | N/A | KPC | NO | N/A |
| Β736 | *K. pneumoniae* | Br sec | KPC | N/A | N/A | KPC | NO | N/A |
| Β804 | *K. pneumoniae* | Br sec | KPC | N/A | N/A | KPC | NO | N/A |
| Β849 | *K. pneumoniae* | Br sec | KPC | N/A | N/A | KPC | NO | N/A |
| Β1002 | *K. pneumoniae* | Br sec | KPC | N/A | N/A | KPC | NO | N/A |
| Β1132 | *K. pneumoniae* | Br sec | KPC | N/A | N/A | KPC | NO | N/A |
| Β1248 | *K. pneumoniae* | Br sec | KPC | N/A | N/A | KPC | NO | N/A |
| Β1430 | *K. pneumoniae* | Br sec | KPC | N/A | N/A | KPC | NO | N/A |
| Β1540 | *K. pneumoniae* | Br sec | KPC | N/A | N/A | KPC | NO | N/A |
| Β1663 | *K. pneumoniae* | Br sec | KPC | N/A | N/A | KPC | NO | N/A |
| B2207 | *K. pneumoniae* | Br sec | KPC | N/A | N/A | KPC | NO | N/A |
| B2221 | *K. pneumoniae* | Br sec | KPC | N/A | N/A | KPC | NO | N/A |
| B2462 | *K. pneumoniae* | Br sec | IN | N/A | N/A | OXA-48 | YES | NDM/OXA-48 |
| B2478 | *K. pneumoniae* | Br sec | IN | N/A | N/A | NDM/OXA-48 | YES | NDM/OXA-48 |
| B2477 | *K. pneumoniae* | Br sec | KPC/MBL | N/A | N/A | KPC/VIM | NO | N/A |
| Β3969 | *P. stuartii* | Br sec | MBL | NEG | N/A | VIM/OXA-48 | NO | N/A |
| Β3461 | *K. pneumoniae* | CVC | MBL | NEG | NEG | VIM | NO | N/A |
| Β840 | *K. pneumoniae* | CVC | KPC | N/A | N/A | KPC | NO | N/A |
| B1289 | *K. pneumoniae* | CVC | KPC | N/A | N/A | KPC | NO | N/A |
| Β1523 | *K. pneumoniae* | CVC | KPC | N/A | N/A | KPC | NO | N/A |
| B2018 | *K. pneumoniae* | CVC | KPC | N/A | N/A | KPC | NO | N/A |
| B2138 | *K. pneumoniae* | CVC | KPC/MBL | N/A | N/A | KPC/NDM | NO | N/A |
| B2244 | *K. pneumoniae* | CVC | MBL | NEG | POS | NDM | NO | N/A |
| B2367 | *K. pneumoniae* | CVC | KPC | N/A | N/A | KPC | NO | N/A |
| B2253 | *P. stuartii* | CVC | MBL | NEG | NEG | VIM | NO | N/A |
| F462 | *K. pneumoniae* | Pleural fluid | KPC | N/A | N/A | KPC | NO | N/A |
| B949 | *K. pneumoniae* | Pus | IN | N/A | N/A | OXA-48 | YES | OXA-48 |
| Η713 | *E. coli* | Rectal swab | MBL | NEG | POS | NDM | NO | N/A |
| H2322 | *E. coli* | Rectal swab | MBL | NEG | POS | NDM | NO | N/A |
| H3303 | *K. pneumoniae* | Rectal swab | MBL | NEG | POS | NDM | NO | N/A |
| H3256 | *K. pneumoniae* | Rectal swab | KPC | N/A | N/A | KPC | NO | N/A |
| Η3295 | *K. pneumoniae* | Rectal swab | KPC | N/A | N/A | KPC | NO | N/A |
| Η66 | *K. pneumoniae* | Rectal swab | MBL | NEG | POS | NDM | NO | N/A |
| Η763 | *K. pneumoniae* | Rectal swab | KPC | N/A | N/A | KPC | NO | N/A |
| **Isolate ID** | **Species** | **Source** | **DMDT** | **HODGE** | **PCR for NDM** | **LFA** | **disagreement** | **AMR** |
| H1003 | *K. pneumoniae* | Rectal swab | IN | N/A | N/A | KPC | YES | KPC |
| H1013 | *K. pneumoniae* | Rectal swab | IN | N/A | N/A | KPC | YES | KPC/NDM |
| H1264 | *K. pneumoniae* | Rectal swab | KPC | N/A | N/A | NEG | YES | KPC |
| Η1546 | *K. pneumoniae* | Rectal swab | KPC | N/A | N/A | KPC | NO | N/A |
| H2002 | *K. pneumoniae* | Rectal swab | KPC/MBL | N/A | N/A | KPC/VIM | NO | N/A |
| H2004 | *K. pneumoniae* | Rectal swab | KPC/MBL | N/A | N/A | KPC/VIM | NO | N/A |
| H2013 | *K. pneumoniae* | Rectal swab | IN | N/A | N/A | OXA-48 | YES | NDM/OXA-48 |
| H2058 | *K. pneumoniae* | Rectal swab | MBL | NEG | POS | NDM | NO | N/A |
| H2095 | *K. pneumoniae* | Rectal swab | KPC | N/A | N/A | KPC | NO | N/A |
| H2121 | *K. pneumoniae* | Rectal swab | IN | N/A | N/A | NDM/OXA-48 | YES | NDM/OXA-48 |
| H2162 | *K. pneumoniae* | Rectal swab | KPC | N/A | N/A | KPC | NO | N/A |
| H2195 | *K. pneumoniae* | Rectal swab | MBL | NEG | POS | NDM | NO | N/A |
| H2257 | *K. pneumoniae* | Rectal swab | MBL | NEG | POS | NDM | NO | N/A |
| H2258 | *K. pneumoniae* | Rectal swab | MBL | NEG | POS | NDM | NO | N/A |
| H2312 | *K. pneumoniae* | Rectal swab | KPC/MBL | N/A | N/A | KPC/VIM | NO | N/A |
| H2347 | *K. pneumoniae* | Rectal swab | KPC | N/A | N/A | KPC | NO | N/A |
| H2273 | *P. stuartii* | Rectal swab | MBL | POS | N/A | VIM | NO | N/A |
| Β3363 | *K. pneumoniae* | Surgical wound | KPC | N/A | N/A | KPC | NO | N/A |
| Β2828 | *K. pneumoniae* | Surgical wound | MBL | NEG | POS | NDM | NO | N/A |
| B4092 | *K. pneumoniae* | Surgical wound | IN | N/A | N/A | OXA-48 | YES | OXA-48 |
| Β721 | *K. pneumoniae* | Surgical wound | MBL | NEG | POS | NDM | NO | N/A |
| Β1210 | *K. pneumoniae* | Surgical wound | MBL | POS | N/A | VIM | NO | N/A |
| A4795 | *E. cloacae* | Urine | MBL | NEG | POS | NDM | NO | N/A |
| A4780 | *K. pneumoniae* | Urine | KPC/MBL | N/A | N/A | KPC/NDM | NO | N/A |
| Α6453 | *K. pneumoniae* | Urine | MBL | NEG | POS | NDM | NO | N/A |
| Α4561 | *K. pneumoniae* | Urine | MBL | NEG | POS | NDM | NO | N/A |
| Α5061 | *K. pneumoniae* | Urine | KPC/MBL | N/A | N/A | KPC/NDM | NO | N/A |
| Α4306 | *K. pneumoniae* | Urine | MBL | NEG | POS | NDM | NO | N/A |
| Α4877 | *K. pneumoniae* | Urine | MBL | NEG | POS | NDM | NO | N/A |
| A7575 | *K. pneumoniae* | Urine | KPC | N/A | N/A | NEG | YES | KPC |
| Α1444 | *K. pneumoniae* | Urine | MBL | NEG | POS | NDM | NO | N/A |
| Α1497 | *K. pneumoniae* | Urine | KPC | N/A | N/A | KPC | NO | N/A |
| Α1481 | *K. pneumoniae* | Urine | KPC | N/A | N/A | KPC | NO | N/A |
| A2080 | *K. pneumoniae* | Urine | IN | N/A | N/A | OXA-48 | YES | OXA-48 |
| Α2164 | *K. pneumoniae* | Urine | MBL | NEG | POS | NDM | NO | N/A |
| Α2640 | *K. pneumoniae* | Urine | KPC | N/A | N/A | KPC | NO | N/A |
| Α2791 | *K. pneumoniae* | Urine | KPC/MBL | N/A | N/A | KPC/VIM | NO | N/A |
| Α2815 | *K. pneumoniae* | Urine | KPC | N/A | N/A | KPC | NO | N/A |
| A2846 | *K. pneumoniae* | Urine | KPC | N/A | N/A | KPC | NO | N/A |
| Α3014 | *K. pneumoniae* | Urine | MBL | NEG | POS | NDM | NO | N/A |
| Α2969 | *K. pneumoniae* | Urine | KPC | N/A | N/A | KPC | NO | N/A |
| **Isolate ID** | **Species** | **Source** | **DMDT** | **HODGE** | **PCR for NDM** | **LFA** | **disagreement** | **AMR** |
| A3836 | *K. pneumoniae* | Urine | KPC | N/A | N/A | KPC | NO | N/A |
| A4109 | *K. pneumoniae* | Urine | KPC | N/A | N/A | KPC/OXA-48 | NO | N/A |
| A4113 | *K. pneumoniae* | Urine | KPC | N/A | N/A | KPC | NO | N/A |
| A4248 | *K. pneumoniae* | Urine | MBL | POS | N/A | VIM | NO | N/A |
| A4236 | *K. pneumoniae* | Urine | KPC/MBL | N/A | N/A | KPC/VIM | NO | N/A |
| A4310 | *K. pneumoniae* | Urine | KPC | N/A | N/A | KPC/VIM | NO | N/A |
| A4423 | *K. pneumoniae* | Urine | KPC | N/A | N/A | KPC | NO | N/A |
| A4451 | *K. pneumoniae* | Urine | IN | N/A | N/A | OXA-48 | YES | OXA-48 |
| A4339 | *K. pneumoniae* | Urine | MBL | N/A | N/A | NDM | NO | N/A |
| A4511 | *K. pneumoniae* | Urine | KPC/MBL | N/A | N/A | KPC/VIM | NO | N/A |
| A4816 | *K. pneumoniae* | Urine | KPC/MBL | N/A | N/A | KPC/VIM | NO | N/A |
| A4807 | *K. pneumoniae* | Urine | IN | N/A | N/A | OXA-48 | YES | OXA-48/NDM |
| A4876 | *K. pneumoniae* | Urine | KPC/MBL | N/A | N/A | KPC/VIM | NO | N/A |
| A4319 | *K. pneumoniae* | Urine | KPC | N/A | N/A | KPC | NO | N/A |
| Α6545 | *P. mirabilis* | Urine | IN | N/A | N/A | NEG | NO | N/A |
| Α7510 | *P. mirabilis* | Urine | MBL | NEG | N/A | VIM | NO | N/A |
| Α2656 | *P. mirabilis* | Urine | MBL | POS | N/A | VIM | NO | N/A |
| Α2966 | *P. mirabilis* | Urine | MBL | NEG | NEG | VIM | NO | N/A |
| A4282 | *P. mirabilis* | Urine | MBL | NEG | NEG | VIM | NO | N/A |
| A4325 | *P. mirabilis* | Urine | IN | N/A | N/A | OXA-48 | YES | OXA-48 |
| Α4505 | *P. stuartii* | Urine | MBL | POS | N/A | VIM | NO | N/A |
| B2155 | *P. mirabilis* | Wound | MBL | POS | N/A | VIM | NO | N/A |

**Table S2.** Carbapenem-resistant *P. aeruginosa* isolates tested for carbapenemase production using the double meropenem disc test (DMDT) and the lateral flow assay (LFA). The Antimicrobial Resistance Direct Flow Chip (AMR) was used in cases of disagreement. Br sec: bronchoalveolar secretions; CVC: central venous catheter; N/A: not applicable; NEG: negative; MBL: metallo-β-lactamase; VIM: Verona integron-encoded metallo-β-lactamase.

| **Isolate ID** | **Source** | **DMDT** | **LFA** | **disagreement** | **AMR** |
| --- | --- | --- | --- | --- | --- |
| D2165 | Blood | MBL | VIM | NO | N/A |
| D18 | Blood | NEG | VIM | YES | VIM |
| D410 | Blood | MBL | VIM | NO | N/A |
| D1432 | Blood | MBL | VIM | NO | N/A |
| D1409 | Blood | MBL | VIM | NO | N/A |
| D1351 | Blood | MBL | VIM | NO | N/A |
| D1854 | Blood | MBL | VIM | NO | N/A |
| D1848 | Blood | MBL | VIM | NO | N/A |
| D182 | Blood | MBL | VIM | NO | N/A |
| D462 | Blood | MBL | VIM | NO | N/A |
| D497 | Blood | MBL | VIM | NO | N/A |
| D797 | Blood | MBL | VIM | NO | N/A |
| D811 | Blood | MBL | VIM | NO | N/A |
| D944 | Blood | MBL | VIM | NO | N/A |
| D1346 | Blood | MBL | VIM | NO | N/A |
| D1349 | Blood | NEG | NEG | NO | N/A |
| D1384 | Blood | MBL | VIM | NO | N/A |
| D1387 | Blood | MBL | VIM | NO | N/A |
| B4064 | Br sec | NEG | NEG | NO | N/A |
| Β2599 | Br sec | MBL | VIM | NO | N/A |
| B3551 | Br sec | NEG | VIM | YES | VIM |
| Β4086 | CVC | NEG | NEG | NO | N/A |
| Β1519 | CVC | MBL | VIM | NO | N/A |
| F501 | Pleural fluid | NEG | VIM | YES | VIM |
| Β1535 | Pus | MBL | VIM | NO | N/A |
| H2273 | Rectal swab | MBL | VIM | NO | N/A |
| B3371 | Soft tissue infection | MBL | VIM | NO | N/A |
| A7124 | Urine | NEG | VIM | YES | VIM |
| Α7609 | Urine | NEG | NEG | NO | N/A |
| Α1351 | Urine | MBL | VIM | NO | N/A |
| Α2953 | Urine | MBL | VIM | NO | N/A |
| Η1649 | Urine | MBL | VIM | NO | N/A |
